# Clinic Atlanta Medical College

**Published:** 1876-02

**Authors:** A. W. Calhoun

**Affiliations:** Professor of Diseases of the Eye and Ear


### Clinic Atlanta Medical College.

A. W. CALHOUN, M.D., Professor of Diseases of the Eye and Ear.

(*Continued from page* 603 *of January Journal*)

Case XXX.—*Occlusion of both Pupils, resulting from Iritis—Restoration of Sight to one Eye by means of an "Artificial Pupil."* (*Iridectomy.*)—J. P., of Eatonton, Georgia, æt. sixty-five, has had several attacks of iritis, causing attachments between the pupil and the anterior capsule of the lens (posterior synechiæ,) and a filling up of the pupillary space by means of an exudative membrane.

Perception of light in the right eye is very slight. With the left he is able to count figures when held quite near him, but is unable to go alone.

Aside from the hope of increasing his vision, an operation upon the iris is justifiable and desirable for the purpose of relieving the constant traction upon the iris, the result of posterior synechiæ, which are an ever present source of recurrent irritis, as demonstrated by the case above.

A large outward iridectomy was made upon the left eye, the artificial pupil exposing a clear portion of the lens, the exudation not having extended further in any direction than the pupillary margin of the iris. The vision of the patient was vastly increased at once. The eye was bandaged and treated in the ordinary way for several days, at the end of which time the patient was able to find his way quite alone, and could even recognise persons with much readiness. An exact test of his vision was not made, but it increased gradually until the eye had thoroughly recovered from the effects of the operation. One or two weeks later, the same operation was made upon the right eye, after which it was found that cataract also existed; but as there were some complications connected with the disease, it was thought best not to resort to any further surgical interference.

CASE XXXI.—*Syphilitic Iritis, with large "Gummy Tumors" upon the Iris* (*Iritis Gummosa.*) *Cure.*—M. C., Atlanta, æt. thirty, presents himself covered with the secondary effects of syphilis, viz., papules and maculæ. From the history of the case, we learn that he had a chancre about three years ago, but supposed himself entirely free from the disease, until the appearance of the maculous and papulous syphilis four weeks since, which now thickly covers the face and body.

Some four weeks ago the right eye became inflamed, but has now of itself nearly recovered. The appearance of the eye indicates the disease to have been a slight attack of irritis, (no doubt syphilitic) of the posterior synechiæ, positive evidence of inflammation of the iris having previously existed, are very clearly distinguishable.

One week ago the left eye began to inflame, and we now find not only all the ordinary symptoms of irritis present, but another symptom, which, in my experience, is invariably indicative of a constitutional syphilitic taint. Jutting out from the anterior surface of the iris, just above the upper portion of the pupillary margin, is a tumor about the size of a small white pea, with a few engorged blood vessels running over it; the contents of these tumors are the same as found in the syphilitic nodules on the sternum or elsewhere—a jelly like substance. They not unfrequently grow to such an extent as very nearly to fill the anterior chamber, and their mode of disappearance is by ulceration or gradual absorption. I have yet to see one in a patient, whose history did not point to a syphilitic trouble, although there may not have been any sign of the disease present at the time, in any other part of the body. As a rule, however, you will find that they occur at the same time with the secondary forms of syphilis.

The patient was put upon constitutional treatment, using, in addition to mercury internally, mercurial inunctions daily, rubbing in one drachm at a time. Locally, atropine (gr. iv. to ℥j) was dropped into the eye every three or four hours; blisters were applied behind the ears, and the patient kept in a dark room, taking occasional mercurial purges, and undergoing the other usual remedies for iritis. The posterior synechiæ were soon broken up, allowing the pupil to become widely dilated, which is an invariable evidence in iritis that you have the disease under control. The *gummy tumor* gradually disappeared, leaving the iris in sound condition, and the secondary effects of the syphilis upon the skin, have very nearly all been removed. The patient was directed to keep up the remedies for some time longer, and dismissed.

Case XXXII.—*Blepharitis and Chronic Conjunctivitis.*—Wm. R., Atlanta, æt. twenty-four, complains of the edges of his lids becoming easily swollen and red. You find that in the lashes are quantities of little scales, and that the lashes fall out quite easily. The cause of this is an inflammation of the follicles and little glands supplying the eye lashes, and this must be cured in order to relieve the unpleasant appearance about the eye lids. The inflammation has also extended into the conjunctiva.

For the blepharitis, we will put the patient upon a salve, composed of the *red oxide mercury, gr. ij, and ointment of rose water,* ʒj., a small piece to be rubbed into the edges of the lids every night on going to bed. For the conjunctivitis, a solution of zinc, alum and morphine, will be found very excellent, to be dropped into the eye several times daily. The disease is very stubborn, and hence the remedies must be kept up longer than there is an apparent necessity for it, for it is prone to return.

Note.—Patient returned several weeks later with no appearance of the disease, but was advised to continue the treatment for one or two months longer.

Case XXXIII.—*Central Opacities of the Corneæ, Obstructing Vision—Iridectomy.*—E. H., Albany, Ga., æt. twenty, had, when a child, purulent ophthalmia in both eyes, resulting in large and thick opacites in the centre of each cornea, reducing his sight to such an extent that he is only able to attend to such duties as require but little use of the eyes. There also exists in the right eye a small central capsular cataract.

A large iridectomy was made to the nasal side of the right eye and behind the clear portion of cornea, which, after healing, notwithstanding the presence of the capsular cataract, has improved his vision to such a degree, that he is now able to prosecute his studies with infinitely more satisfaction than ever before.

Later, the same operation was made upon the left eye, with some improvement of vision, but not to the same extent as in the other eye, possibly on account of a partial atrophy of the optic nerve.

Whether the capsular cataract in the right eye was due also to the purulent ophthalmia, which produced perforation of the cornea, is not certain, but the mother thinks it existed prior to the attack.

---

### SERVICES OF PROF. W. F. WESTMORELAND, M.D.

By T. M. McINTOSH, M.D., Reporter.

*Fibro-Cystic Tumor of the Upper Jaw.*—We have here an immense tumor, which presents the appearance, as you see, of a growth from the under jaw, but which a careful examination proves to be a fibro-cystic tumor of the superior maxillary. He tells us that twelve years ago, or when he was twenty-three years of age (he is now thirty-five), he noticed a small tumor or "rising" in the vicinity of the first molar tooth of right upper jaw. Its growth, for the first eight or nine years, was very slow, but for the last three or four years has been much more rapid—two-thirds of the entire growth has been in the past three years. He has never suffered pain at anytime from the tumor—not so much as an occasional toothache. The only inconvenience for the first few years of its growth was the deformity, and at present the hideous appearance, with the great difficulty of introducing food into the mouth and of mastication is the only inconvenience he suffers. We find that we have almost complete closure of the jaw from the growth overlaping the under jaw and filling almost the entire buccal cavity, thus preventing mastication. The history we have had in this case is the usual history in this affection—a slow and continuous growth of the bone without pain, or other inconvenience, except which may arise from the size of the tumor. In some cases we have an occasional toothache from a displaced tooth, or an occasional neuralgia from the compression or stretching of the nerve, differing radically in these particulars from that affection (encephaloid cancer), which, in appearance, it so much resembles.

If we examine this growth more carefully, we find an expansion of the bone, and at some points the expansion is so great that the bone is not thicker than a sheet of paper, and by pressure with the finger we may make indentation, and a sound similar to that produced by pressing parchment. This immense mass, then, is the expanded superior maxillary with cysts, containing a fluid in greater or less quantity, fibrous tissue, of more less consistence—sometimes almost as consistent and dense as a fobroid. The only remedy that promises anything for this poor man is the complete oblation of this entire mass—the removal of the left superior maxillary. If he can stand the shock of removal, he will be entirely relieved, as the disease is not malignant, and will not return. I have operated in twelve cases, and have never had a return in a single case, so that we may safely promise a cure if he recovers from the immediate effects of the operation.

We find our patient not only willing to have the operation performed, but is desirous that it should be done at once, even after being fully informed of the dangers attending such operations.

The patient was now placed partially under the influence of ether, and an incision made from the angles of the mouth to near the temporo-maxillary articulation, and the soft parts dissected up from the osseous mass. A terrible hemorrhage was the result of this dissection, requiring the ligation of fifteen or twenty arteries or arterioles, and the free application of muriated tinct. of iron to stay the bleeding. After dissecting up the soft parts, a section of the bone was made with the chisel and mallet, and the mass removed. In three weeks he was sufficiently recovered to return to his home in the northeastern portion of this State.

*Hydrocele.*—J. H. M., age 27 years. Here is an enlargement of the testicle, which we have demonstrated to be hydrocele of the sac. Our manner of determining the nature of this disease, and differentiating it from other diseases of the testis, is by the translucent appearance of the testicle when seen by transmitted light. The manner of doing this you have seen on many other occasions, so I will not trouble you by a repetition of the process.

Hydrocele may be of two kinds: hydrocele of the tunica vaginalis testis, and encysted hydrocele. In the first variety the entire serous covering of the testis is filled by a serous, straw-colored fluid, constituting a true dropsy of the sac. In the encysted variety the fluid is formed in a distinct cyst connected with the testicle, pushing the tunica vaginalis testis before it.

The causes of this disease are obscure, most of them having the same history as this case, who says that some months ago he noticed a gradual increase in the size of this testicle, with some little tenderness on pressure over the Epididymis. Sometimes we have a hydrocele from acute orchitis; yet in most instances, when the inflammation which produced the effusion of serum has subsided, the fluid is absorbed; so that these cases which we are most frequently called upon to treat are chronic in character and of similar history to the one before you.

The treatment of hydrocele is either palliative or curative: palliative when the fluid is repeatedly evacuated by puncturing the sac with either the needle, bistoury or trocar. The curative treatment, or the radical cure, as it is sometimes called, consists in drawing off the accumulated fluid with the trocar and canula, and injecting into the sac, through the canula, remaining in the puncture, by a syringe adapted to it, any irritating fluid, as port wine, solution of the sulphate of zinc, tincture of iron, and tincture of iodine, of various strengths; the object being to excite a sufficient amount of inflammation in the sac to regulate the secretion and absorption, so that the fluid naturally secreted in the sac, to lubricate the parts, will not be formed faster than it can be absorbed, and not by causing adhesion of the sides of the sac, and its obliteration, as has been supposed. The seton has also been a very general means adopted for the radical cure of hydrocele, but now the most popular plan is with the tincture of iodine, and in this case I shall use it diluted with an equal portion of water, adding a little of the iodide of potassium. I will inject two or three ounces of this solution into the sac, and allow it to remain until it causes considerable pain along the course of the spermatic cords and in the loins, and then allow it to flow away.

It is well to warn you always to let the canula remain until you have thrown in your injection, for if you do not it will be difficult to introduce the nozzle of the syringe into the cavity of the sac, and you may throw your injection into the cellular tissue of the scrotum, thinking you are into the sac, and cause inflammation and sloughing of the tissues. In every instance the above treatment will not suffice for a permanent cure, because a sufficient amount of inflammation is not excited to regulate the secretion and absorption above spoken of, or the effusion which always results from the inflammation excited by the injection may not be absorbed, though in the great majority of instances this plan of treatment will effect a cure.

*Secondary Syphilis.*—Here we have a case of secondary syphilis, which you remember we prescribed for several months ago. He then had in every feature a perfect type of the secondary form of this disease; such as the eruptions over the face and body, sore throat, alopecia, mucus patches, etc. Under treatment he improved very much, and when you last saw him all the above symptoms had disappeared. The prescription, you remember, was the following:

℞.—Blue Mass, . . . . . . ʒj.

Calomel, . . . . . . . grs. iv.

Opium Pulv., . . . . . . grs. iv.

M. ft. Twelve pills. S. Take one night and morning.

Had he continued his visits regularly he would probably have been cured; but all the symptoms having disappeared, he ceased to come, and now you see his situation—equally as bad as when first seen.

Ricord, the great syphilographer, contended a quarter of a century ago that syphilis, in its severest and most virulent forms, could never be thoroughly eradicated from the system; that even after all the symptoms had disappeared, and the patient apparently completely restored to health, that he would always, even after the lapse of a number of years (twenty or thirty, perhaps), be liable to a reappearace of the disease, with all the virulency that it had shown when it made its first appearance; and if such a person were to marry and have offspring, the children would be liable to be affected by the disease, or would be puny, scrofulous, and of low vitality.

My experience, however, leads me to a different conclusion, for I have treated many cases—some of them in this city—that I can now recall, who had the disease in its most virulent form, that have since married and had children, who are now grown, and neither the children nor the parents have ever exhibited any sign of the return of the disease, nor any symptoms whatever of a scrofulous or tuberculous character, and are as robust and vigorous as children born of parents neither of whom had ever had syphilis. In one instance, I remember, where the husband, with the secondary form of the disease, impregnated his wife, whom I treated as for syphilis, hoping that by impressing her system with anti-syphilitic remedies, I might through this medium impress the fœtus also, and prevent the birth of a child with constitutional syphilis; and in accordance with my anticipations, when the child was born I was much gratified to see a baby as healthy and as large as if no such disease had ever existed, and it has never subsequently shown any of the symptoms or constistitutional manifestations that have been considered as resulting from a syphilitic origin.

I hope by this case to impress you with the reason that I assign why so many cases of syphilis, apparently cured, have had all the symptoms to return, after a longer or shorter period, in an unmodified form, for you remember that the sore throat, eruptions, alopecia, etc., had all disappeared when this man was last here; and he seeing that all the local symptoms had disappeared, and not realizing the importance of further treatment, ceased his visits, with, as you see, a return of all his former symptoms in as equally severe a form as originally.

Often it is the case that this early discontinuance of the anti-syphilitic remedies is by the advice of the physician, who is misled in the same manner as was this man; and of course the disease will return, because it is not cured, and such instances as these contribute to support the belief, so widely prevalent, that syphilis can only be suppressed for a time, not permanently and effecually eradicated from the system.

*Whitlow.*—P. C., age twenty-four years. This woman is here to-day with an affection of the finger, called paronychia, or whitlow; by the unprofessional, bone-felon.

There are four varieties of this disease, named from the character of the tissue involved: the cutaneous, phlegmonous, thecal, and periosteal. This case before you is a typical one of the first variety of this disease, and is now in a suppurating condition, the pus having dissected up the scarf skin to quite the first articulation, and would, perhaps, continue to do so, were it not prevented by the proper treatment, which is to remove the scarf skin separated by the pus from the true skin below. A simple puncture of the skin would not, in many instances, suffice, for the small opening made by the pin would speedily close, and the pus re-accumulate, necessitating a frequent repetition of the puncturing in order to effect a cure.

After having removed the entire separated scarf skin, as you see me do with a small pair of scissors and a tenaculum, there can, of course, be no re-accumulation of the pus. After this is done we simply protect the tender part from air, dust, and other foreign bodies, by a small bit of isinglass plaster.

In your works on surgery you will only find two varieties of whitlow mentioned: that affecting the soft tissues and that affecting the hard parts, for the reason, as they contend, that it is impossible to differentiate them, and, for practical purposes, unnecessary. However, you can diagnose them, at least in the beginning, and it is important that they should be discriminated, for the depth to which the inflammation has extended will be the guide to the extent of an incision in the treatment.

In the cutaneous variety the pain is not so severe, lancinating, tensive, or deep-seated as in the other varieties, the skin having a very red, glazed, shining appearance; and the power of moving the finger without great additional pain is had. In the phlegmonous variety, which involves the cellular tissue, the redness is not so deep; the skin not so tense; the pain more deep-seated, with a thickened, doughy feel of the part. In the thecal the prominent feature is the very intense pain darting up the course of the tendon whenever the slightest motion is made, because of the inflammation existing in its sheath, with the symptoms of the preceding varieties in a less prominent form. In the periosteal variety, where the periosteum is inflamed, the pain is unmistakeable, having that peculiar throbbing, lancinating character, that attend inflammation of the periosteum in other situations, with very little redness of the skin until it has reached the point of suppuration.

It is a peculiariarity of this disease to attack more frequently the first phalanges of the fingers, though it is not invariably in this situation, as it may involve the entire hand. Its causes are obscure, though it is in most instances dependent upon a constitutional cause similar to that of erysipelas, manifesting itself, locally, by an inflammation erysipelatous in character; for in every case of whitlow, particularly of the deeper-seated varieties, there is considerable constitutional disturbance, as restlessness, thirst, and fever. And besides, it often occurs as an epidemic, more frequently in the spring and fall.

For the treatment, little can be done to arrest their development, for the tendency of the disease is to inflammation and sloughing of the tissues, more particularly in the deeper-seated varieties, often leading to extensive destruction of the soft and hard parts, with permanent stiffness of the finger, leaving them in a rigid and extended condition when sloughing of their tendons has occurred. In some instances, in the periosteal variety, the phalanges will necrose, requiring their removal, which should be done, when only the first phalanx is necrosed, through the pulp of the finger, which will leave a member little injured, either in appearance or usefulness.

The treatment in all the varieties will be to give early a brisk purge of some saline cathartic, and to make early, free and bold incision into the tissues as deep as the inflammation extends, for any local treatment will be of but little avail in preventing the development of the disease and modifying its intensity. In making your incision you should always do it from the proximal to the distal extremity, for if the patient jerks he will assist rather than hinder you in making the operation. After this is done, poultice the finger for a few days, and it will speedily get well.

